# Systematic prediction of drug resistance caused by transporter genes in cancer cells

**DOI:** 10.1038/s41598-021-86921-9

**Published:** 2021-04-01

**Authors:** Yao Shen, Zhipeng Yan

**Affiliations:** 1 Department of Computer Science, New Jersey Institute of Technology, Newark, NJ USA; 2 Martin Tuchman School of Management, New Jersey Institute of Technology, Newark, NJ USA

**Keywords:** Biomarkers, Computational biology and bioinformatics, Chemical biology, Cheminformatics

## Abstract

To study the drug resistance problem caused by transporters, we leveraged multiple large-scale public data sets of drug sensitivity, cell line genetic and transcriptional profiles, and gene silencing experiments. Through systematic integration of these data sets, we built various machine learning models to predict the difference between cell viability upon drug treatment and the silencing of its target across the same cell lines. More than 50% of the models built with the same data set or with independent data sets successfully predicted the testing set with significant correlation to the ground truth data. Features selected by our models were also significantly enriched in known drug transporters annotated in DrugBank for more than 60% of the models. Novel drug-transporter interactions were discovered, such as lapatinib and gefitinib with ABCA1, olaparib and NVPADW742 with ABCC3, and gefitinib and AZ628 with SLC4A4. Furthermore, we identified ABCC3, SLC12A7, SLCO4A1, SERPINA1, and SLC22A3 as potential transporters for erlotinib, three of which are also significantly more highly expressed in patients who were resistant to therapy in a clinical trial.

## Introduction

During recent decades, the volume, velocity, and variety of cancer-related data have exploded thanks to new technologies, such as next-generation genomic sequencing and high-throughput screening. This includes large-scale screening of drug sensitivities or gene silencing/knock-out across cancer cell lines as well as molecular characterization of cancer cell lines such as gene expression, somatic mutations, and copy number variations^1^. In addition to cell line models, large institutional efforts have enabled comprehensive molecular profiling of clinical patient samples across different tumor types. For example, the Cancer Genome Atlas (TCGA) project has generated large-scale transcriptional, genomic and epigenomic data for more than 11,000 patients^2^. Integration of these omics data using mathematical models and machine learning techniques will provide insights into various cancer research problems, such as drug resistance.

Recent advances in cancer research have contributed significantly to more effective treatment and improved patient survival. However, in many cases, patients initially respond to therapy but later develop resistance, which leads to disease recurrence and patient relapse. Cancer drug resistance remains a major challenge in medical oncology^3,4^. Drug resistance is a complex problem and can occur by different mechanisms, including alteration of drug targets, drug activation, cell death inhibition, and cancer stem cell populations, as well as alteration of drug efflux and uptake caused by changes in drug transporters.

Drug transporters are membrane proteins that transport a variety of compounds across membranes, including the solute carrier protein (SLC) family and the ATP-binding cassette (ABC) family^5^. The SLC and ABC transporter superfamilies contain transmembrane efflux proteins that translocate structurally diverse substrates, including lipids, metabolites, amino acids, ions and many cancer drugs, across membranes either for uptake or export. Many transporters are involved in multidrug resistance in tumor cells when they are overactivated in cancer cells and export cancer drugs outside the cells, which will lead to tumor resistance^5^. For example, there are 48 ABC genes reported in humans, and they are known to play an important role in mediating the multidrug resistance (MDR) in cancer patients, where patients develop resistance not only to the drugs they are taking but also to other types of drugs^6^. There have been extensive studies on several transporters. For example, ABCB1 (also called p-glycoprotein) is frequently amplified in multidrug-resistant cells and can export various xenobiotics not limited to cancer drugs^7^. Nonsynonymous as well as synonymous single nucleotide polymorphisms (SNPs) of multiple transporter genes have also been found to be associated with interindividual differences in responses to chemotherapy in cancer^8^. Because of sequence similarities among the transporter genes, they are expected to have similar transporting functions. However, there have been limited systematic studies of all transporter genes in cancer cells. A study of ABC transporter genes using NCI60 cancer cell line data was previously reported but mainly focused on the relationship between one transporter and one drug^9^.

In this work, we used a systematic and data-driven approach that applies various machine learning models to study the potential interactions of transporters with drugs that may cause drug resistance in cancer cells. We hypothesized that the difference between the effects of the drug treatment and the silencing of the drug’s primary target in the same cell lines is mainly due to the resistance mechanism in which the drug is transported outside the cells, which reduces the concentration of drugs in the cells. Based on this hypothesis, we built machine learning models using the transcriptional and genomic profiles of all potential transporters to predict viability differences between the drug treatment and the silencing of the drug’s primary target across the same cell lines.

For this, we leveraged multiple public data sets, including drug sensitivities across a panel of cancer cell lines, the gene expression and genetic profiles in the corresponding cell lines, and the genome-wide shRNA or CRISPR-cas9 silencing of individual genes in these cell lines. We split each data set into training and testing sets. We used cross-validation (CV) techniques to select the best parameters when training the models in the training set and evaluated model performance using the testing set. We also trained models on one data set and tested the models with other independent data sets. More than 50% of the models successfully predicted the ground truth data. Features selected by the models were also significantly enriched in known transporters reported in the DrugBank database^10^. We also predicted novel potential transporter drug interactions that may confer drug resistance. The predicted novel transporters for erlotinib were further validated using an in vivo data set of lung cancer patients. Three out of the five predicted transporters were also significantly more highly expressed in the resistant versus the sensitive patients, which indicates their potential roles in drug resistance in patients.

## Results

### Preprocessing of public data sets for analysis

To perform this study, we leveraged multiple publicly available data sets. Drug sensitivity across cancer cell lines has been profiled and deposited in multiple databases, including the CTD2 data set from the Cancer Target Discovery and Development Network (https://ctd2-dashboard.nci.nih.gov/dashboard/)^11^ with 481 compounds across 664 cancer cell lines, the PRISM data set from the Cancer Dependency Map (https://depmap.org/portal/)^12^ with 4516 compounds across 578 cancer cell lines, and the GDSC data set from the Genomics of Drug Sensitivity in Cancer (https://www.cancerrxgene.org/)^13^ with 396 compounds across 986 cell lines. Transcriptional and genomic profiles of cancer cell lines have been sequenced and deposited in the CCLE (Cancer Cell Line Encyclopedia https://portals.broadinstitute.org/ccle)^14^ database (921 cancer cell lines) and the COSMIC (Catalogue of Somatic Mutations in Cancer https://cancer.sanger.ac.uk/cosmic) database^15^ (624 cancer cell lines). Genome-wide shRNA or CRISPR-cas9 knockdown experiments have been performed and are hosted in the DepMap (Cancer Dependency Map, 18333 genes across 625 cancer cell lines) and CERES (Sanger CRISPR data https://score.depmap.sanger.ac.uk/downloads) databases^16^ (17,799 genes across 318 cell lines).

**Figure 1:**
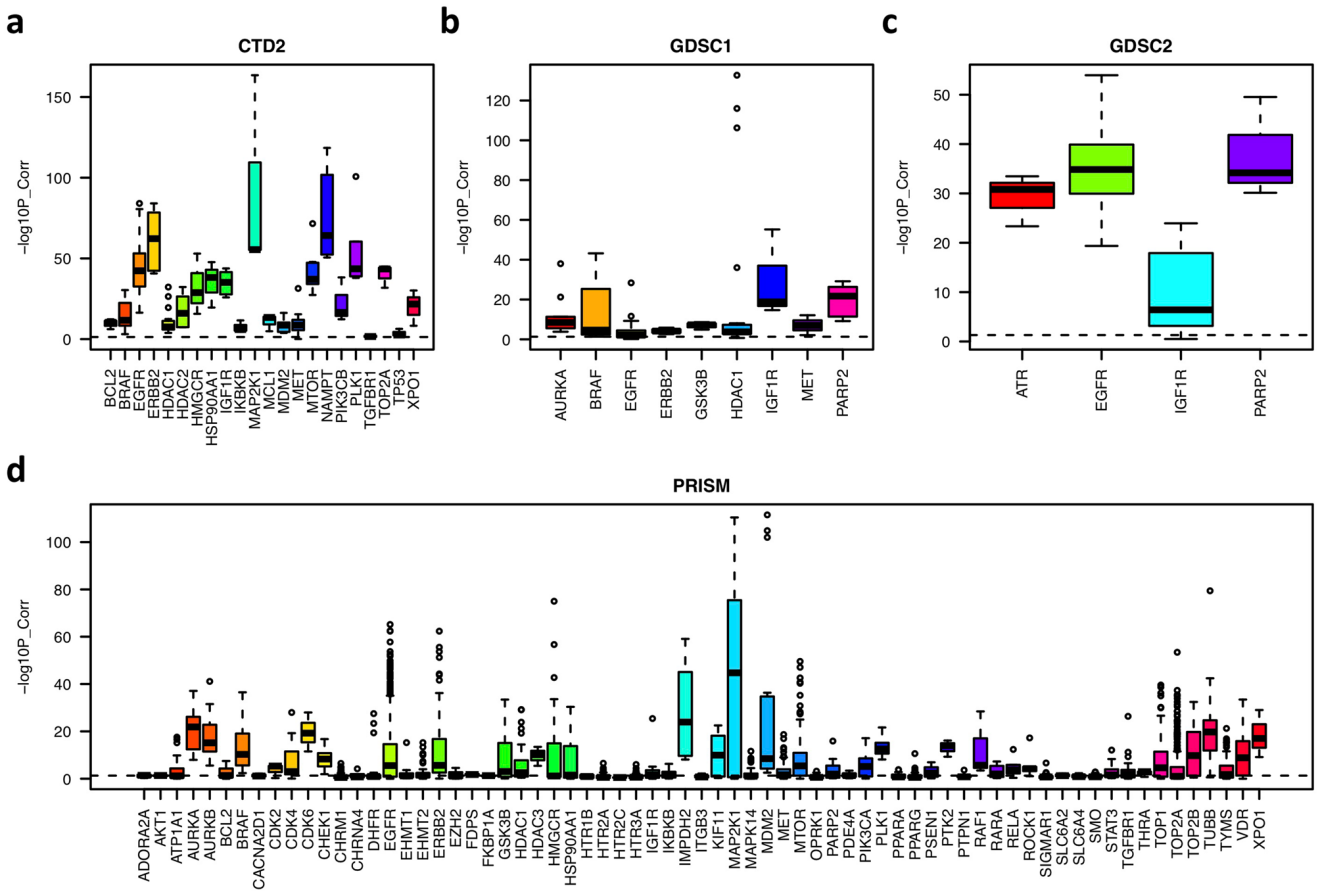
Boxplot of pairwise drug similarity scores targeting the same target in each data set. The similarity score is represented by the negative log10 p-value of the pairwise correlations computed based on their sensitivities across cancer cell lines.

To prepare these data sets for analysis, we performed multiple preprocessing steps. First, we limited the analysis to cell lines that have drug sensitivity screening, transcriptional or genetic profiling, and shRNA or CRISPR silencing data. All these filtering steps are necessary for building machine learning models to predict transporter and drug interactions. Second, we focused our analysis on drugs that only have one or two primary targets. This is to remove drugs with polypharmacological characteristics whose effects in cells could not be recaptured by knocking down a single target. Third, for drugs with the same target, we expected that they share similar sensitivity profiles across cancer cell lines. To test this, we computed their pairwise similarity scores based on their sensitivities across cancer cell lines. As shown in Fig. 1, for different targets, similarities among their drug sets have different distributions. Drugs with targets such as EGFR, ERBB2, MTOR, PARP2, AURKB, MAP2K1 and PLK1 share more similar profiles, which indicates that the inhibition of these targets has specific effects on the in vitro viability and proliferation of cancer cells. However, this is not true for other targets, e.g., HTR1B, TGFBR1, and ADORA2A, whose drugs do not share high similarity. Thus, we removed drugs that were significantly different (correlation p < 0.05) from the other drugs sharing the same target. If a target had fewer than three associated drugs, we also removed it from our analysis.

**Figure 2:**
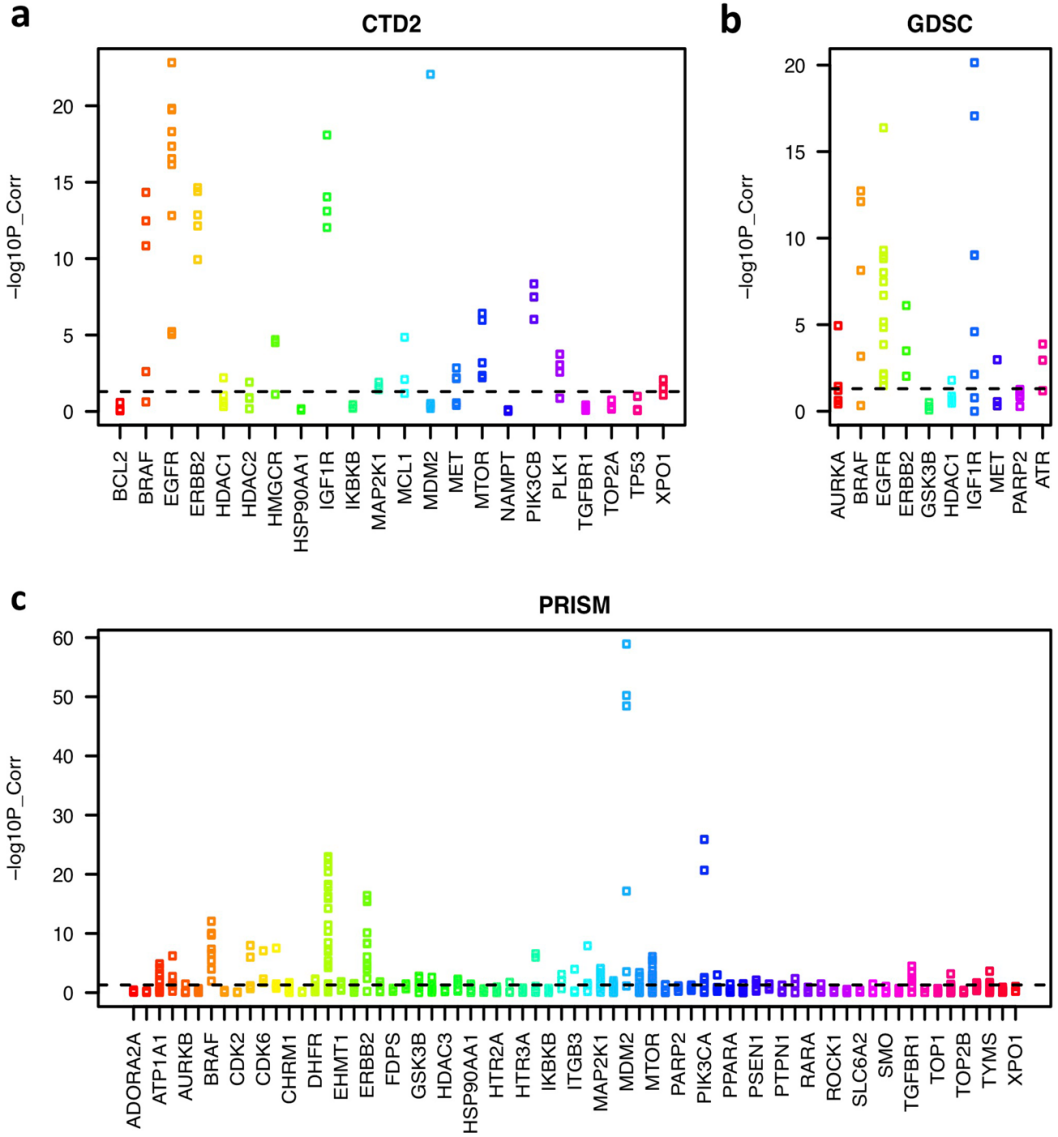
Strip charts of the correlation between cell viabilities upon drug treatment and the silencing of the primary target across the same cancer cell lines. The y-axis represents the negative log10 p-value of the correlation.

We expect that drug treatment should induce similar effects in cells as the silencing of its primary target. For this, we calculated the correlation between the cell viabilities upon drug treatment and the silencing of its primary target across the same cell lines. Some drug treatments and their target silencing showed strong correlations, such as EGFR, ERBB2, IGF1R, MTOR, PLK1, BRAF, AURKB, CDK4, CDK6, and MDM2 inhibitors, which indicates that they cause similar effects on cell proliferation and survival. However, the correlation was low for some other drugs, such as ADORA2A, CHRM1, and IKBKB inhibitors (Fig. 2). The discrepancy may have been caused by multiple factors, for example, off-target effects from the silencing experiments, the polypharmacological features of drugs, no effects of these drugs on in vitro cell survival or proliferation, or noise in the data set. Thus, we focused on drugs whose sensitivities had a significant correlation (p < 0.05) with the silencing of the target.

As a result, we obtained three cell line data sets with complete profiles of drug sensitivity, transcription or genetics, as well as gene silencing viability. The CTD2 data set had a total of 79 unique drugs with sensitivities across 351 cancer cell lines that involve 22 targets, such as EGFR, HDAC1, MTOR, MET, ERBB2, and BRAF. The Prism data set had a total of 537 unique drugs with 69 targets and their sensitivities across 332 cell lines. The targets with the highest number of drugs included EGFR, TOP2A, MTOR, MET, PPARG, and MAPK14. The GDSC data set had a total of 47 unique drugs with ten targets, including EGFR, IGF1R, HDAC1, PARP2, AURKA, and BRAF, as well as their sensitivities across 224 cancer cell lines. The complete list of potential drug transporters was downloaded from the DrugBank database. Any genes that are labeled as transporters or carriers for any drug in DrugBank were included in our models as predictive features.

### Lineage-specific expression of transporter genes

**Figure 3:**
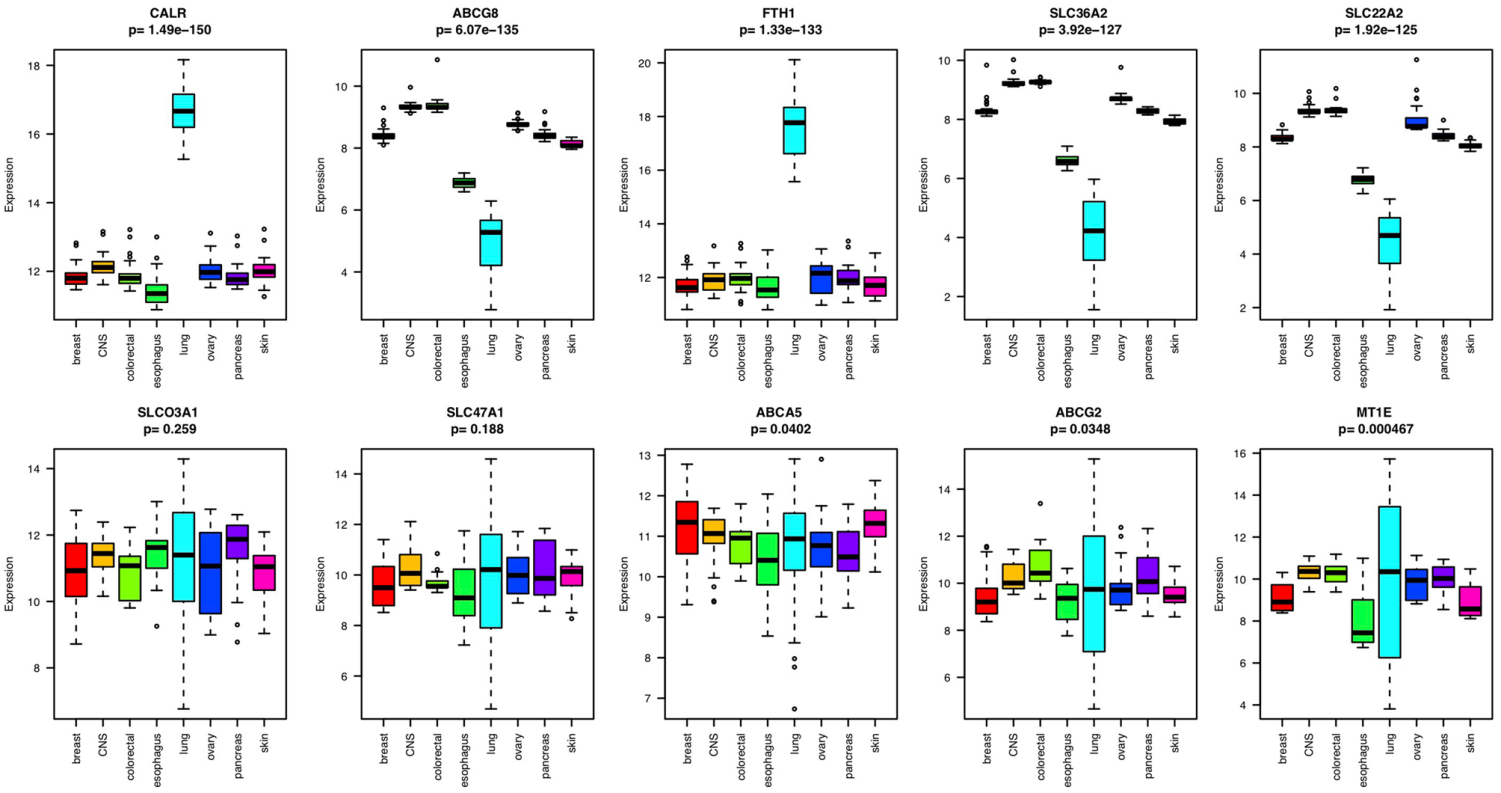
Boxplot of the gene expression of the ten transporters that showed the strongest or weakest lineage-specific distribution in eight different tissue types, each with more than ten cell lines.

To explore the lineage-specific expression of transporters, we generated a heatmap of cancer cell lines from the CCLE database based on the transcriptional profiles of drug transporters. This includes 246 cell lines from eight lineages each with more than ten cell lines. As we expected, cell lines formed clusters of different lineages because of the lineage-specific expression patterns of the drug transporters (Supplementary Figure S1). Among the 262 transporters or carriers, only two of them (SLCO3A1 and SLC47A1) did not show lineage-specific distribution according to ANOVA (p-value > 0.05). ABCA5, ABCG2, MT1E, ABCA1, and CACNB1 also did not have strong lineage specificity. CALR, ABCG8, FTH1, SLC36A2, and SLC22A2 were among the most strongly differentially expressed in different lineages according to ANOVA (p-value < 2e−16). Among the eight lineages, transporters showed more different expression patterns in lung and esophageal tissues than in other tissue types (Fig. 3).

### Construction of machine learning models to predict the difference between drug treatment and its target silencing caused by the overactivation of drug transporters

We hypothesized that the difference between the drug treatment and the silencing of its target was mainly caused by the overactivated drug transporters that transport drugs outside the cells, which reduces the drug concentrations in the cells and induces drug resistance. To test this hypothesis, we built biomarker models based on the transcriptional and genetic profiles of all potential transporters to predict the difference between the drug sensitivities and the sensitivities of the silencing of the primary target across cancer cell lines (i.e., DiffSen).

For each drug within each data set, we randomly split the data into a 70% training set and a 30% testing set. Then, using the training set, we built models based on the transcriptional and genetic profiles of all potential transporters to predict DiffSen. For feature selection, we computed the correlation of each feature (i.e., the predictive variables) with DiffSen (i.e., the target variable) and selected the top features with high correlation values to be used in the models. We trained multiple regression models, including Lasso, Ridge, and Random Forest, with a five-fold CV technique to select the best parameters for each model. Model performance was evaluated by computing the correlation between the predicted values and DiffSen (the ground truth data). The final predictions for the test set were obtained from the predictions using the model with the best performance in the training set.

Among the 49 models built from the CTD2 data set, the predicted values had a significant correlation with DiffSen (i.e., the ground truth data, p-value < 0.05) in the testing set for 27 drugs. Eleven out of 33 models for the GDSC data set and 84 out of 171 models for the Prism data set successfully predicted the ground truth data in the testing set (correlation p-value < 0.05). The reason why the CTD2 and Prism data set had better performance than the GDSC data set may be that the gene expression profiles of CTD2 and Prism were sequenced using the RNASeq platform, whereas the GDSC data set was obtained using microarrays to measure gene expression, which may be less accurate than using RNASeq data.

**Figure 4:**
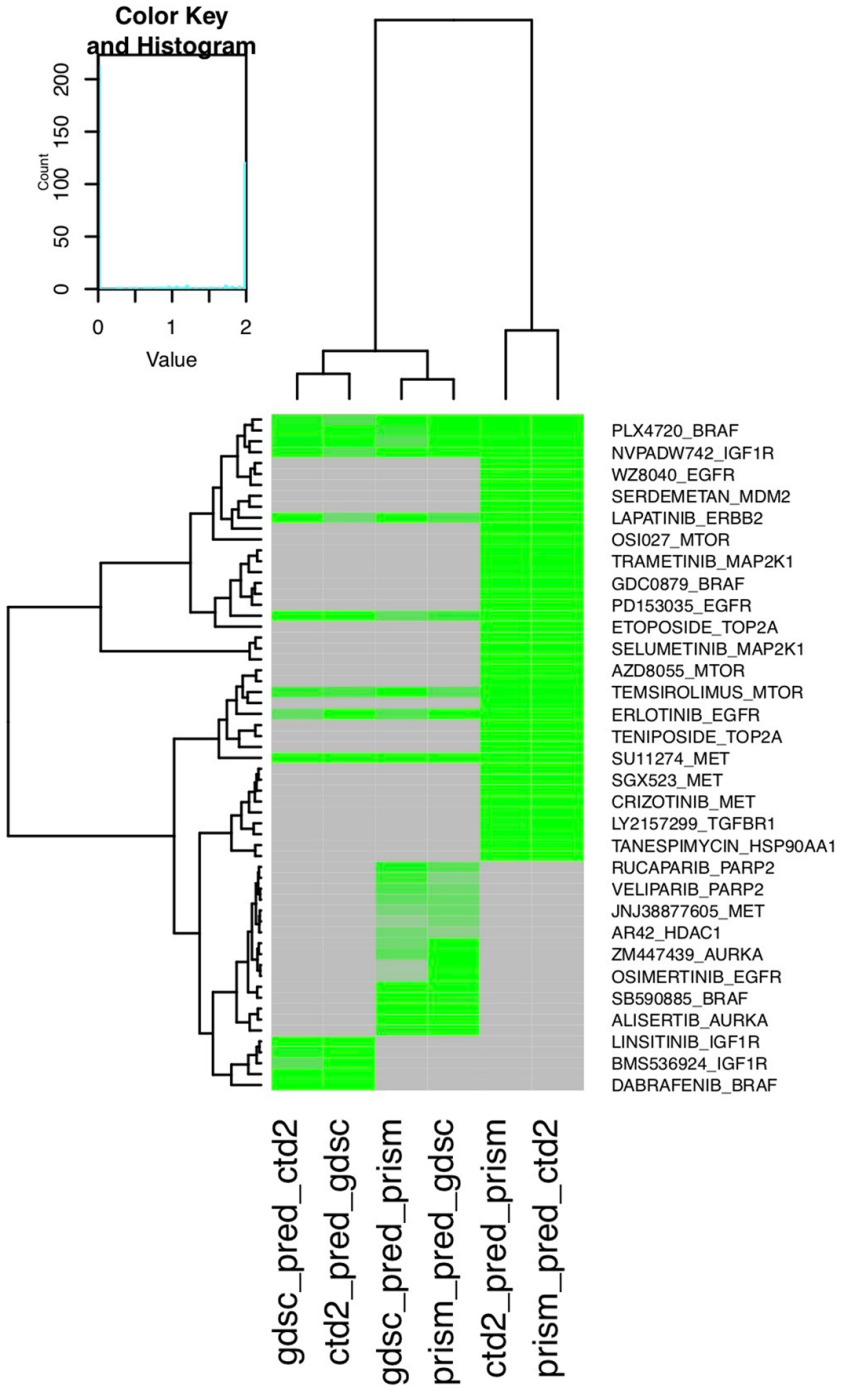
Heatmap showing model performance using one data set as the training set and another data set as the testing set. Model performance is represented by the negative log10 p-value of the correlation between the predictions versus the ground truth data. Gray indicates that the model is not available; i.e., the drug does not exist in either data set. Models with a more intense green colors have better performance.

Next, we tested whether the models built from one data set could also predict the DiffSen for the same drug in another data set. For example, we used the CTD2 data set as the training data to tune the parameters and build models. Then, we used the model with the best performance for the training set to make predictions for the testing set, i.e., the GDSC and the Prism data sets. Similarly, for each model, we selected the top features that had the highest positive correlation with DiffSen across the same cell lines in the CTD2 set. Overall, 47 out of 78 models successfully predicted the ground truth data (correlation p-value < 0.05) when using one data set for training and another independent data set for testing (Fig. 4). These models included gefitinib, afatinib, lapatinib, and erlotinib targeting EGFR; linsitinib, BMS754807, and NVPADW742 targeting IGF1R; PLX4720 and dabrafenib targeting BRAF; and temsirolimus targeting MTOR (Fig. 4).

### Enrichment of the predictive features in known transporters

**Figure 5:**
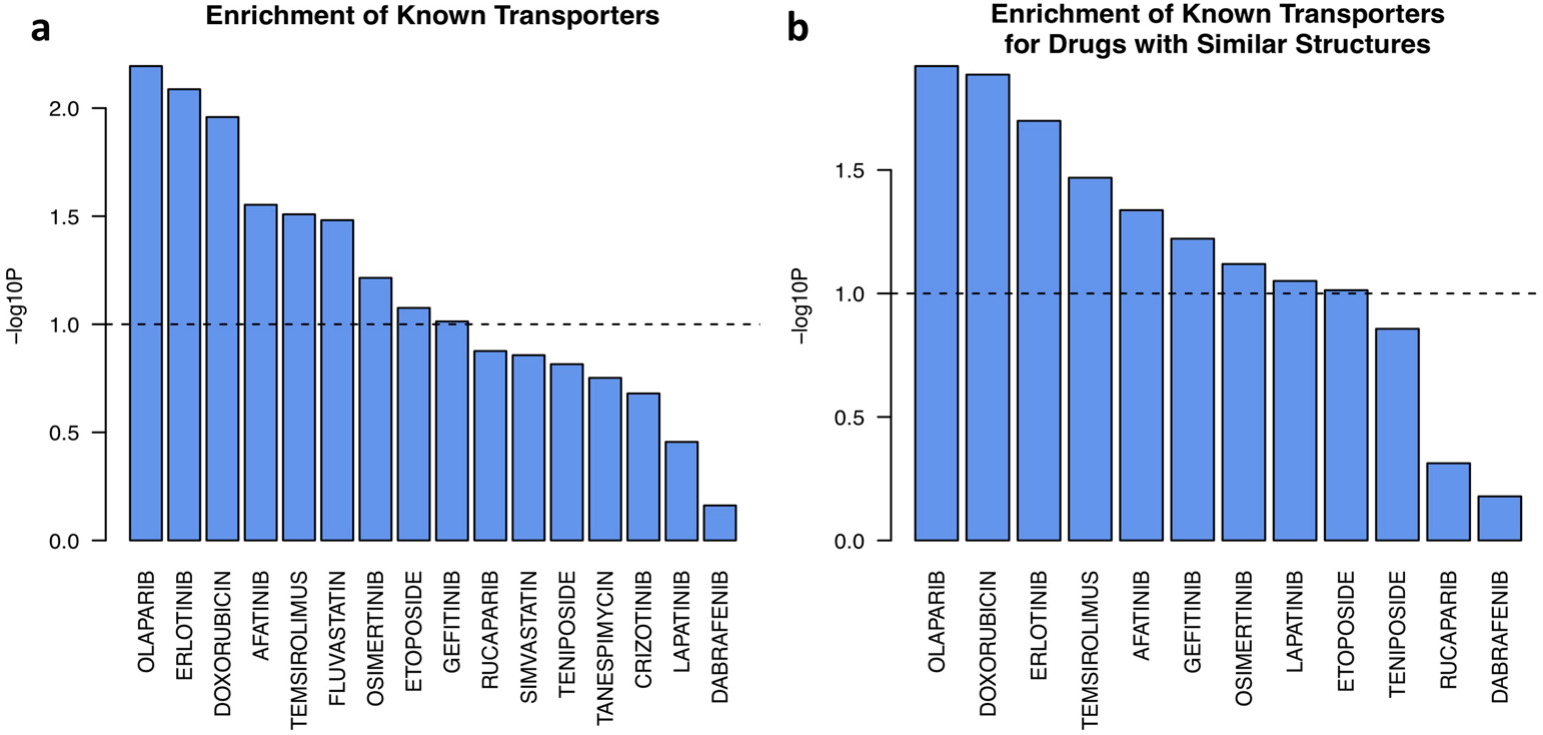
Enrichment of known transporters on the integrated list of all potential transporters sorted by their correlation with DiffSen. The predictive features are integrated across data sets for successful models of (**a**) the same drug, or (**b**) drugs with similar structures.

Among the models that successfully predicted the ground truth data in another data set, we expect that the predictive features used by the models are potential transporters that induce drug resistance. For this, we obtained transporters reported for drugs from the DrugBank database and tested whether the annotated transporters were enriched in the predictive features we selected. Thus, for each drug with successful models (p-value < 0.05 for the correlation between the prediction and the ground truth data) in any of the three data sets, we first rank sorted all the features based on their correlation with DiffSen and then integrated the rank lists across all models for the same drug by taking the average. Then, we computed the enrichment of the reported transporters for the specific drug obtained from DrugBank on the sorted list of all transporters. Among all 16 drugs with transporter information in DrugBank, the annotated transporters for six of them were significantly enriched (p < 0.05) in the predictive features, and three additional drugs had borderline significant enrichment (p < 0.1, Fig. 5).

Furthermore, we expected that drugs with similar structures would bind to similar transporters. To test this, we integrated the predictive features of drugs with similar structures (Tanimoto coefficient based on 2D descriptors > 0.35). There were a total of 12 drugs in the data sets whose structures were similar to those of some other drugs, for which we integrated their predictive features. The known transporters for nine of them had significant enrichment in the predictive features with a high positive correlation with DiffSen (p < 0.05, Fig. 5). Thus, for each feature, we computed its overall importance score for every drug after integration with its structurally similar drugs. This score implies its potential role as a transporter for the specific drug.

There were multiple features whose overexpression was strongly associated with the resistance of multiple drugs, such as ABCC3, SLC4A4, ABCA1, SLC7A7, SLC22A3, POU5F1, SCNN1A, AKR1C3 and ABCG2. For each of these potential transporter genes, we also calculated the enrichment of the top ten cell lines with the highest or lowest expression on the list of cell lines sorted by the DiffSen of a drug-target pair. Supplementary Figure S2 summarizes the enrichment analysis results for these features as well as their correlation with DiffSen in individual data sets. Some of the potential novel interactions include lapatinib and gefitinib with ABCA1, olaparib and NVPADW742 with ABCC3, and gefitinib and AZ628 with SLC4A4, where these transporters may induce resistance to the corresponding drugs.

There were multiple EGFR inhibitors in our data set, including erlotinib, gefitinib, and afatinib, with highly similar structures (Tanimoto coefficient > 0.35). For them, we integrated their predictive features and identified the most common features, including ABCC3, SLC12A7, SLCO4A1, SERPINA1, and SLC22A3. Supplementary Figure S3 shows the correlation and the enrichment analysis of these five transporters with erlotinib in the GDSC data set.

**Figure 6:**
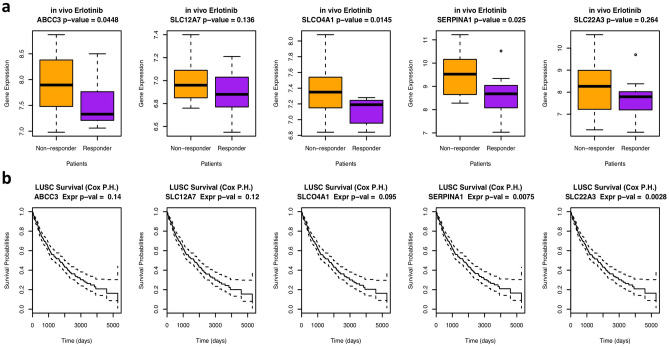
The expression values of the five potential transporters are different in the responsive vs. resistant patients and can predict patient survival. (**a**) Boxplot showing the difference in the expression values of the five potential transporters in the responsive vs. resistant patients in an erlotinib clinical trial (GSE33072). The p-value was computed from Student’s t-test by comparing the gene expression in responders versus nonresponders. (**b**) Cox proportional hazard survival analysis showing how expression values of different genes affect patient survival in the TCGA lung cancer data set, with p-values illustrating the significance of the analysis.

To test whether these five transporters are also potential transporters in cancer patients, we analyzed a publicly available data set (GSE33072) for a clinical trial of erlotinib in lung cancer patients^17^. The patients were classified into responders and nonresponders depending on whether their survival time was greater than eight weeks. We performed Student’s t-test to compare the gene expression values between the responders and the nonresponders and found that all five transporters had higher expression in the resistant patients than in the sensitive patients (median expression value). Three of them were significantly overexpressed in the resistant patients (p < 0.05, Fig. 6), which implies their potential role as erlotinib transporters that induce drug resistance in patients. We also tested whether these five genes are related to lung cancer patient survival by analyzing the TCGA lung cancer data set using the Cox proportional hazard model. Two of the genes displayed a significant association with patient survival (p < 0.05), and one showed borderline significance (p < 0.1).

## Discussion

There are a few data-driven studies examining the associations between transporters’ gene expression levels and drug resistance across cancer cell lines. For example, a pioneering study analyzed the correlation of 48 ABC transporters expression levels with 1429 cancer drug sensitivities in the NCI60 cell line panel. It revealed and experimentally validated specific transporters that confer drug resistance^9^. Similar research has also been performed on the SLO and SLC22 transporter families where the overexpression of SLC22A4 in cell lines were experimentally verified to rescue the sensitivity of mitoxantrone and doxorubicin^18^. Another research group has applied a LASSO regression model to predict drug resistance based on the transcriptional and genomic profiles of transporters in cancer cells based on the GDSC drug sensitivity data set^19^. However, these studies have been limited by the types of multi-omics data sets analyzed as well as the various machine learning models applied.

In this work, we successfully built multiple machine learning models based on the transcriptional and genetic profiles of transporters to predict the difference between cell viabilities upon drug treatment and the silencing of the target. Predictions from the testing set were significantly correlated with the ground truth data for more than 50% of the models. For example, three out of the five known transporters and carriers of erlotinib, including ABCB1, SLCO2B1, and ABCG2 are predicted among the top 10% of all potential transporters in our analysis. We also identified for the first time the transporters that interact with multiple drugs, such as ABCC3, SLC4A4, and ABCA1. More specifically, we identified ABCC3, SLC12A7, SLCO4A1, SERPINA1, and SLC22A3 as candidate transporters for erlotinib. Three of these are also significantly overexpressed in cancer patients who are resistant to therapy compared with sensitive patients. Some of these transporters have been reported to be associated with the resistance to tyrosine kinase inhibitors (TKIs) or chemotherapy, e.g., SLC12A7 has been discovered to be up-regulated in the TKI-resistant cell lines^20^, ABCC3 and SLCO4A1 have been shown as markers for chemotherapy outcome in lung cancer patients^21–23^, the differential expression of SERPINA1 has also been reported to be associated with chemoresistance in human epithelial ovarian cancer^24^.

There are several limitations of this study. First, when performing cross-data set analysis, we had to use features that were profiled and shared in both data sets. This limited the features to mostly gene expression with several genetic features. As a result, we might have missed mutations or copy-number variations in the transporters that cause drug resistance. In addition, we expected that the overactivated transporters would transport drugs outside the cells and thus reduce the drug concentrations in the cells. However, we built models mainly based on the transcriptional profiles of the transporters. The expression levels of transporters do not always correlate with the protein activities of the transporters due to the gene-wise differences in the transcriptional or translational rates, post-translational modifications, protein stability and many other factors. However, large-scale proteomics experiments that measure protein activities are often not available. Instead, computational methods, e.g., the VIPER algorithm, which, when integrated with tumor regulatory or signaling networks, can accurately infer context-specific protein activities in cancer cells^25^. This suggests that to incorporate such virtually inferred transporter protein activities in future analysis could potentially improve our models. Second, drug mechanisms of resistance may be different among lineages. Thus, it may be helpful to build models that focus on cell lines from the same tissue type for drugs with lineage-specific mechanisms. Third, we expected that the difference between cell viabilities upon drug treatment and silencing of the target was due to drug transporters. Thus, our approach would not work for drugs with polypharmacological effects, such as kinase inhibitors, whose effect could not be captured by silencing an individual gene.

Overall, this is the first time that various types of large-scale data sets, i.e., drug sensitivities, drug chemical structures, cell line genetic and transcriptional profiles, and genome-wide gene silencing experiments, have been systematically integrated to study transporter-induced drug resistance in cancer cells. We expect that these findings will be of great value for understanding the drug resistance problem caused by transporters and help design combination therapies to overcome drug resistance.

## Materials and methods

### Cell line data sets

We obtained drug sensitivity data sets from the Cancer Target Discovery and Development Network (https://ctd2-dashboard.nci.nih.gov/dashboard/), the Cancer Dependency Map (https://depmap.org/portal/), and the Genomics of Drug Sensitivity in Cancer (https://www.cancerrxgene.org/). We downloaded cancer cell line transcriptional and genomic profiles from the Cancer Cell Line Encyclopedia (https://portals.broadinstitute.org/ccle) and the Catalogue of Somatic Mutations in Cancer (https://cancer.sanger.ac.uk/cosmic). We obtained genome-wide shRNA or CRISPR-cas9 knockdown data sets from the DepMap (https://depmap.org/portal/) and the CERES databases (https://score.depmap.sanger.ac.uk/downloads).

### Machine learning methods to build predictive models

We built various machine learning models, including Random Forest^26^, Lasso Regression, and Ridge Regression models^27^, based on the transcriptional and genetic profiles of the drug transporters to predict DiffSen. Random Forest is an ensemble learning method that constructs a multitude of decision trees from randomly sampled subsets of training samples and makes predictions by averaging or performing majority voting on the outputs of all trees. We tuned parameters to optimize the classifiers based on the training set. There are two important parameters to which random forest models are sensitive. One is the number of variables available to split in each node. The other is the number of trees to grow. We performed a grid search to obtain the best parameters that minimized the out-of-bag error rate. We used the randomForest package in R to perform this analysis. Lasso and Ridge regression are both regression methods that apply regularization techniques to reduce the size of the coefficients and overcome overfitting. The difference between Lasso and Ridge is that Lasso applies the L2 penalty while Ridge applies the L1 penalty to the coefficients. The lambda parameter needs to be tuned for both types of models, and it was selected using cv.glmnet in the glmnet package in R.

To obtain an unbiased estimate of classifier accuracy while controlling for overfitting, we performed five-fold CV for model training. Five-fold CV is an iterative process that first randomly splits the training samples into five equal folds, then builds models on four folds, and finally makes predictions on the one fold left out for testing. This process is repeated five times, and hence, all training samples are tested after five iterations. For feature selection, we selected the top features whose values had the highest positive correlation with the dependent variable across cancer cell lines. Model performance was evaluated by computing the correlation between the predicted values and the ground truth data.

### Drug structure similarity calculation

We first downloaded the SMILES representation of the drug structures from the DrugBank database (https://www.drugbank.ca/) or the PubChem database (https://pubchem.ncbi.nlm.nih.gov/) for a total of 10,028 compounds and then converted it to the SDF format using the chemmineR package in R. Then, drug structure similarity was computed based on the SDF format using the same package, which computes the Tanimoto coefficient based on the 2D descriptors.

### Analyzing the in vivo erlotinib patient data set

We downloaded the Battle clinical trial data set (GSE33072) from the GEO database, which contains the RNASeq profiles of the lung cancer patients prior to erlotinib treatment with patient response data for a total of 131 patients. The raw RNASeq data were normalized using DESeq2 software in R^28,29^. Student’s t-test was performed to compare gene expression levels in the resistant patients versus the sensitive patients.

### Survival analysis of the TCGA lung dataset

The lung cancer patient dataset was downloaded from the TCGA database with annotations of whether the patient relapsed and their survival time for a total of 420 lung cancer patient samples. For the transporter genes that we were interested in, we performed Cox proportional hazard survival analysis to test whether the expression levels of these genes had any effect on patient survival.

## Supplementary Information

Supplementary Information.
