# Supplementary material for: Systematic prediction of drug resistance caused by transporter genes in cancer cells
Source: Sci Rep. 2021 Apr 1;11:7400. doi: 10.1038/s41598-021-86921-9 (PMC8016963; doi:10.1038/s41598-021-86921-9)
Supplement: Supplementary file 1 — Supplementary Information. [file 41598_2021_86921_MOESM1_ESM.docx]

**Supplementary Information**

**Systematic prediction of drug resistance caused by transporter genes in cancer cells**

Yao Shen^1*^, Zhipeng Yan^2^

^1^Department of Computer Science, New Jersey Institute of Technology, Newark, New Jersey

^2^Martin Tuchman School of Management, New Jersey Institute of Technology, Newark, New Jersey

*To whom correspondence should be addressed:

Department of Computer Science

New Jersey Institute of Technology

Newark, NJ

07102

Phone: 937-596-3366

Email: yshen@njit.edu


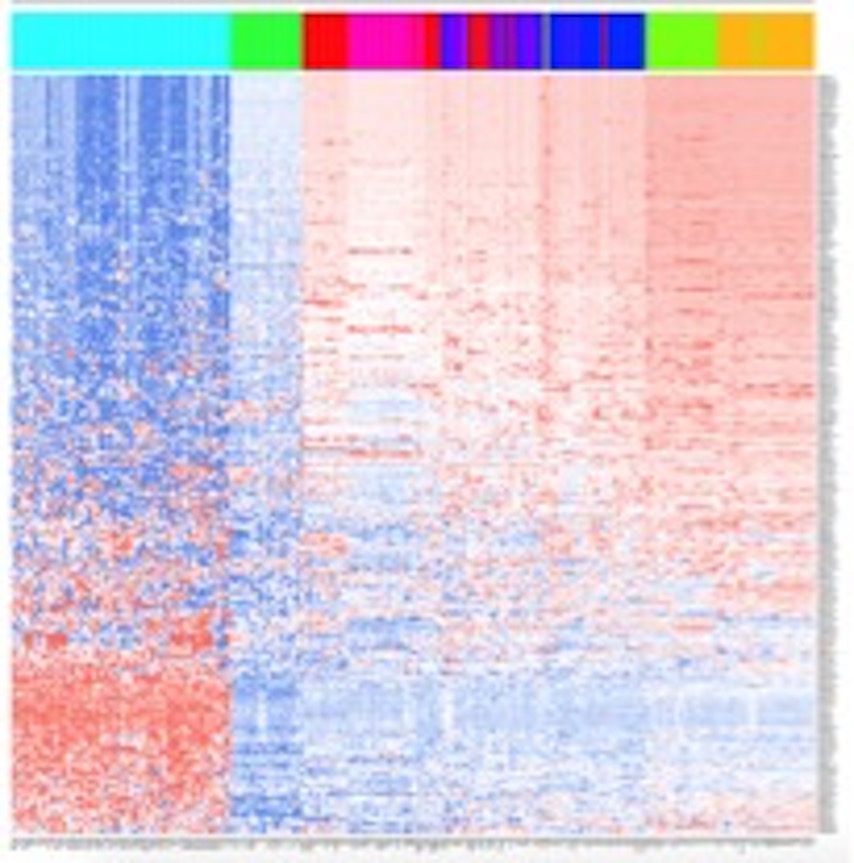


**Supplementary Figure 1:** Heatmap of the cancer cell lines from the CCLE database based on the transcriptional profiles of the drug transporters. Blue, white, and red colors indicate low, intermediate and high gene expression. Side color indicates different tissue types.


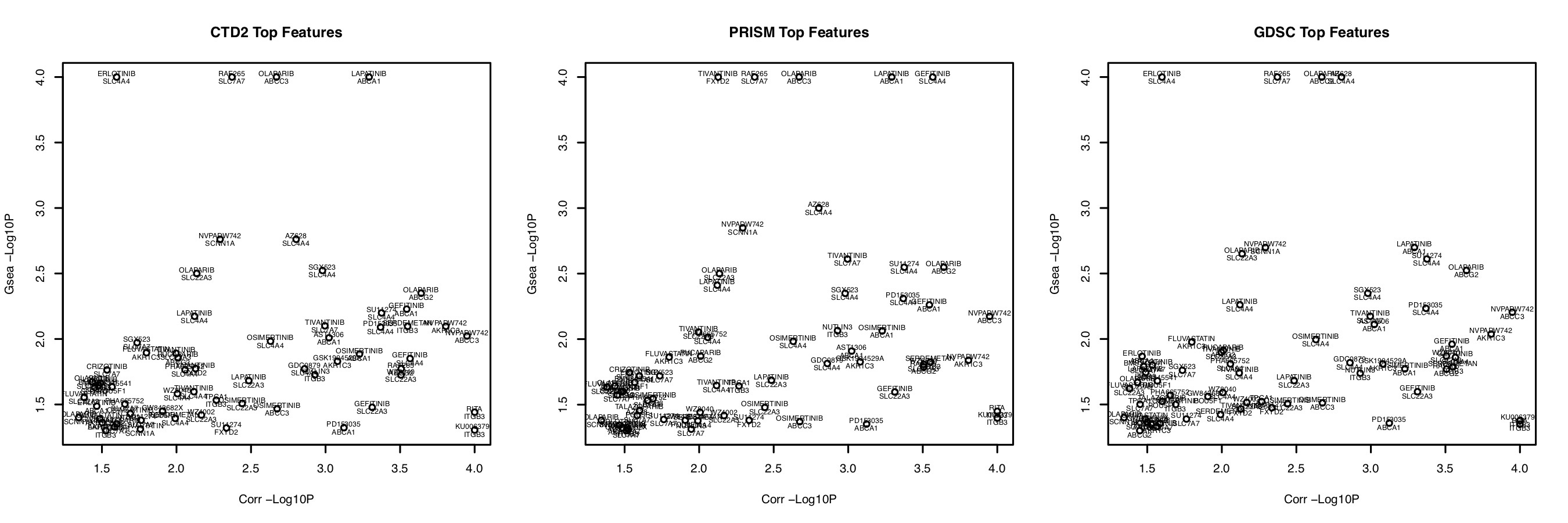


**Supplementary Figure 2:** Correlation and enrichment analysis reveals common genes as potential multiple drug transporters. Each point represents a drug-target pair. The x-axis represents the negative log10 p-value of the correlation between transporter expression and DiffSen. The y-axis represents the negative log10 p-value of the enrichment of the top 10 cell lines with the highest or lowest transporter gene expression on the list of all cell lines sorted by DiffSen.


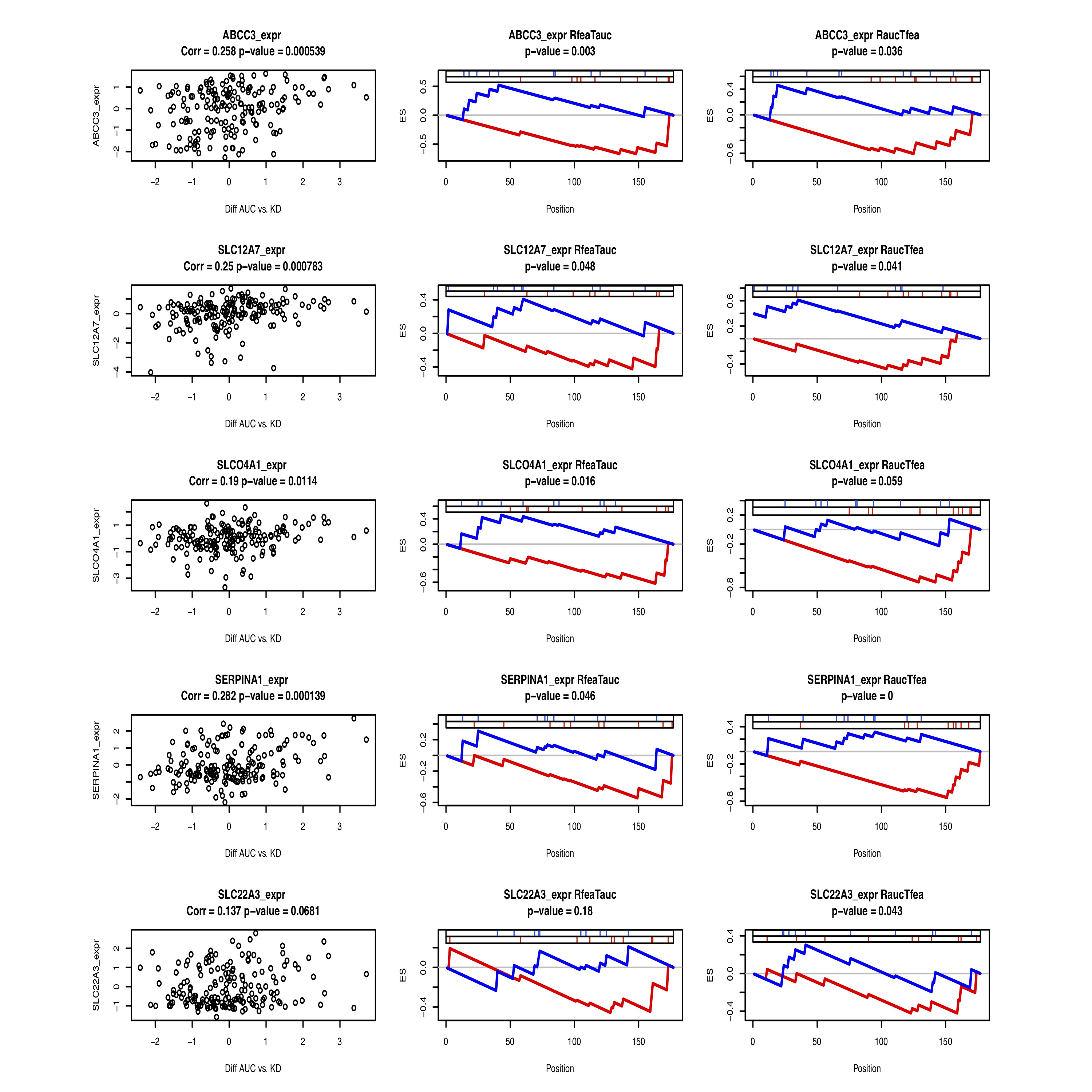


**Supplementary Figure 3:** Relationships between the top 5 features with DiffSen for erlotinib in the GDSC data set. Left: correlation between the expression values of each of the five transporters with DiffSen. Right: enrichment of the top 10 cell lines with the most overexpressed or repressed transporters on the list of all cell lines sorted by DiffSen.
